# The Efficacy of CO_2_ Vaginal Laser in the Treatment of Recurrent, Post-Coital and Interstitial Cystitis: A Multicentric Prospective Study

**DOI:** 10.3390/jcm13123550

**Published:** 2024-06-17

**Authors:** Daniela Luvero, Adele Silvagni, Anna Maria Angioli, Maurizio Filippini, Francesco Plotti, Roberto Montera, Carlo De Cicco Nardone, Erika Notaro, Francesco Branda, Roberto Angioli

**Affiliations:** 1 Unit of Gynecology, Fondazione Policlinico Universitario Campus Bio Medico, Via Alvaro del Portillo 200, 00128 Roma, Italy; 2 Research Unit of Gynecology, Department of Medicine and Surgery, Università Campus Bio Medico, Via Alvaro del Portillo 21, 00128 Roma, Italy; 3 Department of Obstetrics and Gynecology, Hospital State of Republic of San Marino, 47893 Borgo Maggiore, San Marino; 4 Unit of Medical Statistics and Molecular Epidemiology, Università Campus Bio Medico, Via Alvaro del Portillo 21, 00128 Roma, Italy

**Keywords:** CO_2_, laser, daily pollakiuria, dysuria, urgency, interstitial cystitis, post-coital cystitis, recurrent cystitis, vaginal atrophy, hyaluronic acid

## Abstract

**Background**: This multicentric prospective study was carried out at Fondazione Policlinico Universitario Campus Bio Medico and Ospedale di Stato of St. Marino Republic. Between 1 January 2019, and 31 December 2022, all pre- and post-menopausal women diagnosed with recurrent, post-coital, and interstitial cystitis at both centers were included in the study. The main aim of the study was to assess the effectiveness of vaginal CO_2_ laser treatment, alone or combined with intravesical hyaluronic acid instillations, in managing cystitis symptoms, such as dysuria, pollakiuria, and urgency, across the entire patient cohort. The secondary objective was to investigate the reduction in number of annual cystitis episodes post-treatment. **Methods**: Each woman underwent three to four sessions of micro-ablative CO_2_ vaginal laser treatment. A follow-up examination was conducted 12 months after the final laser session (up to December 2023), during which a post-treatment VAS assessment evaluated dysuria, daily pollakiuria, and urgency. The enrolled patients recorded the number of cystitis episodes experienced during the 12-month pre- and post-treatment period. **Results**: Results indicated the laser’s efficacy in reducing the total number of cystitis episodes per year and an improvement in symptoms up to one year post-treatment. Greater efficacy of the CO_2_ laser treatment, particularly when combined with intravesical hyaluronic acid instillation, was observed in both pre- and post- menopausal women. **Conclusions**: Fractional CO_2_ laser therapy represents a safe and efficacious, non-hormonal approach for pre- and post-menopausal women diagnosed with recurrent, post-coital, and interstitial cystitis.

## 1. Introduction

Worldwide, recurrent urinary tract infections (rUTI) in women significantly contribute to both morbidity and healthcare expenses. It is estimated that 25% of women of all ages will experience a recurrence of UTI, with an almost 50% lifetime risk of acquiring one [1]. A recurrent urinary tract infection (r-UTI) is characterized by symptoms that endure following the resolution of a prior UTI; these occurrences typically manifest twice within a span of 6 months or three times within a year [2]. Lower urinary tract symptoms, which often have identifiable causes, are characterized by sterile urinalysis and urine cultures, along with bladder, urethral, or diffuse pelvic pain collectively referred to as the ‘bladder disease complex’. Pain may be limited to the pelvic organs, pelvic floor myofascial support, or external genitalia, presenting hallmark symptoms of chronic pain alongside urinary symptoms [3]. Interstitial Cystitis–Bladder Pain Syndrome (IC–PBS) constitutes a component of this complex [4]. According to the International Continence Society (ICS), IC–BPS is characterized by suprapubic pain related to bladder filling, along with other urinary symptoms such as dysuria, urgency to void, frequency, increased daytime and night-time frequency, all in the absence of a confirmed urinary infection or other obvious pathology [5]. As IC–BPS is still regarded as a diagnosis of exclusion, it is often identified belatedly, or misdiagnosed [6]. The univocal etiology of IC–BPS remains unclear, and currently, there is no effective treatment available for achieving remission and complete recovery from the disease [7]. Analgesics, lifestyle changes, and patient education are recommended as first-line treatments for IC–BPS. Second-line therapies include intravesical infusion therapy (using dimethyl sulfoxide, antibiotics, heparin, or hyaluronic acid), and submucosal intravesical injection with botulinum toxin type A. However, these treatments are thought to provide symptom relief for only a limited duration. This multiparametric approach underscores the importance of tailoring the treatment strategy for IC–BPS to address the individual needs of each patient [8]. IC–PBS can present as recurrent non-bacterial cystitis or non-bacterial post-coital cystitis, depending on the number of episodes per year or the triggering factor determining the onset of urinary symptoms. Recurrent cystitis refers to a cohort of patients experiencing reduced quality of life, encountering cystitis episodes at least twice in the preceding 6 months or at least three times in the preceding 12 months [9,10]. Post-coital non-bacterial cystitis precisely refers to a cystitis episode occurring 24–72 h after sexual intercourse [11,12]. During sexual intercourse, the male partner can act as a “passive” instrument, facilitating the inoculation and spread of uro-pathogenic microorganisms through the urethral meatus. In non-bacterial cystitis, it has been hypothesized that the real cause may be the low positioning of the urethral meatus proximal to the vaginal opening, brought on by increased friction from penile thrusts during sexual activity [13]. In consideration of the complex multi-parametric and personalized approach to the treatment of IC–PBS that often occurs, even within the framework of urogenital menopausal syndrome, the present study aims to investigate the role of CO_2_ micro-ablative vaginal laser therapy in the management of urinary symptoms. The CO_2_ vaginal laser exploits a technology that has been utilized in gynecology for over 30 years, offering minimally invasive treatment for a broad range of vulvar, vaginal, and cervical conditions. In 2014, the FDA approved fractional CO_2_ lasers for application in gynecology and aesthetic medicine, dermatology, and plastic surgery, for procedures including incision, excision, ablation, vaporization, and coagulation of soft body tissues [14]. Over the last decade, there has been a rapid increase in the adoption of vaginal CO_2_ laser treatment for conditions including the genitourinary syndrome of menopause (GMS), menopausal or iatrogenic vulvar and vaginal atrophy, vulvo–vaginal lichen sclerosus, vulvar or vaginal intraepithelial neoplasia, and uro–gynecological disorders such as overactive bladder syndrome and stress or mixed urinary incontinence [15,16,17,18,19,20]. Focusing on uro–gynecological pathologies, a significant multicenter prospective study was published by Ogrinc UB involving 175 women with urinary incontinence to assess the potential of vaginal erbium YAG laser as a treatment approach for SUI and MUI. Results from the one-year follow-up indicate that, while the vaginal erbium YAG laser can efficaciously be considered an effective minimally invasive treatment strategy for stress urinary incontinence (SUI), its efficacy for mixed urinary incontinence (MUI) appears to be comparatively lower [21]. In other studies, the use of CO_2_ vaginal lasers, in comparison to the non-laser groups, demonstrated significant improvements in sexual function and a reduction in symptoms related to atrophy (dryness, dyspareunia, burning and itching sensation, tendency to vaginitis and lower urinary tract infections) among post-menopausal women affected by GMS; however, these studies often had a limited sample size and brief follow-up period [22,23,24,25,26,27]. In 2023, the EUGA Working Group conducted a comprehensive review wherein the obtained data suggest that both non-ablative erbium YAG and micro-ablative CO_2_ vaginal lasers are safe energy-based therapeutic strategies for the management of symptoms related to vaginal atrophy and genitourinary syndrome of menopause in post-menopausal women and breast cancer survivors that experienced iatrogenic menopause [28]. Similar results regarding the safety and efficacy of diode laser, were demonstrated by Figerio et al. in a 2023 prospective study conducted on a population of menopausal women with contraindication for the use of hormone replacement therapy, local or systemic. The data demonstrate high tolerability, efficacy in the treatment of genitourinary symptoms, and easy applicability in an outpatient setting [29].

The CO_2_-based lasers exploit a technology suitable for structural regeneration that is often utilized to address micro-lesions in superficial tissues. Specifically, the fractional CO_2_ laser penetrates deeper layers to trigger collagen and extracellular matrix production, facilitating tissue trophism recovery [30,31]. Water proves to be an excellent medium for absorbing the frequency of laser light emitted at 10,600 nm. Mucosal tissues are practically vaporized to stimulate collagen synthesis, given water’s significant presence within these tissues [32]. The rationale for utilizing this technology is to precisely promote neo-collagenase, facilitating the migration of new collagen to the mucosal surface. This leads to an increase in fibroblast activity and in the fibrillar component of the extracellular matrix. These effects are translated into a significant increase in the thickness and glycogenic load of the vaginal epithelium, aimed at alleviating symptoms such as vaginal dryness, vaginal dyspareunia, vaginal tightness, prolapse symptoms, and improving bladder function, urgency, and stress incontinence [33,34,35,36]. A 2023 prospective study conducted by Perona et al. investigated the cellular and tissutal modifications in response to vaginal fractional-pixel-CO_2_ laser treatment. Among the collected histological samples, an improvement in cellular proliferation (evidenced by Ki67 staining) was noted, as well as an increase in telomere length and in collagen, and hyaluronic acid and elastic fibers levels inside the epithelial lamina propria. All these demonstrate that the laser induces a restoration of the vaginal epithelium [37]. Another retrospective study conducted in 2024 by Yueming et al. investigated the effects of CO_2_ laser therapy in modifying vaginal bacterial flora in women suffering from urogenital menopausal syndrome, showing that both the CO_2_ micro-ablative vaginal laser and estrogen therapies can regulate the vaginal microbiota imbalance of GSM and improve the corresponding symptoms [38].

The main aim of this prospective multicentric study was to evaluate the efficacy of the CO_2_ vaginal laser in the treatment of urinary symptoms, specifically for dysuria, urgency and daytime pollakiuria, in a population of pre- and post-menopausal women suffering from interstitial, recurrent, and post-coital cystitis. The interest of the present study is therefore mainly focused on urinary symptoms, which are the symptoms most frequently associated with the above-mentioned disorders. A decision was made to neglect from the investigation additional symptoms associated with vaginal atrophy and urogenital syndrome of menopause, which have been extensively explored in recent studies in the scientific literature. In particular, it was decided to also include women at a fertile age in order to evaluate the effectiveness of the vaginal laser on a subpopulation that is poorly taken into consideration in current studies in the scientific literature but is extremely involved, from an epidemiological point of view, by the above-mentioned pathologies.

The secondary objective was to evaluate the efficacy of the treatment in decreasing the number of cystitis episodes per year. Additionally, the study aimed to investigate whether concurrent therapies, such as intravesical instillations of hyaluronic acid, and the presence or the absence of vaginal atrophy, may impact the efficacy of the CO_2_ laser. The scientific rationale for performing a specific investigation on these two subpopulations was based on two different reasons, as follows. The first was to look for a direct correlation between the reduction in vaginal atrophy severity due to laser-induced tissue regeneration and the improvement in the urogenital symptoms under investigation. The second was to find out whether the effects of the vaginal laser are cumulative compared to the treatment obtained with the addition of intravesical instillations of hyaluronic acid for the management of urogenital symptoms and the reduction in cystitis flare-ups per year.

## 2. Materials and Methods

This bicentric prospective study was carried out at Fondazione Policlinico Universitario Campus Bio Medico of Rome and Ospedale di Stato of St. Marino Republic. All pre- and post-menopausal women diagnosed with recurrent, post-coital, and interstitial cystitis between 1 January 2020 and 31 December 2022 were recruited from both centers. All enrolled patents signed an informed consent to be enrolled in the study, which was approved by the local ethical review board.

The inclusion criteria were the following: age > 18 years, negative urinalysis for infectious cystitis at the time of enrollment, negative urine cytology for neoplastic cells, post- coital cystitis (cystitis occurring between 24 and 72 h after sexual intercourse), recurrent cystitis (more than 3 episodes of cystitis per year) and interstitial cystitis diagnosed via histological samples obtained by biopsy during operative cystoscopy.

The exclusion criteria were the following: previous urological or gynecological surgery, previous or ongoing urinary tract neoplasms, previous pelvic radiation, ongoing genital infections, genital prolapse stage higher than 2 according to the Pelvic Organ Prolapse Quantification System, urinary tract abnormalities, poorly controlled diabetes mellitus, hypertension and psychiatric disorders, urinary stress/urge incontinence, pregnancy, alcohol or drug addiction, ongoing therapy with diuretics, β3-adrenergic and anticholinergic drugs. The concomitant use of hyaluronic acid bladder instillations or local administration of hyaluronic acid cream were not listed as an exclusion criterion. In order to avoid bias in the assessment and in the characterization of symptoms, all patients with a urogenital anatomy altered by previous surgery and radiotherapy, by current infectious or oncological pathologies, by systemic syndromes or medications that directly or indirectly affects urination and diuresis, were excluded. For the same reason, it was decided to exclude all patients with genetic or acquired functional and morpho-structural abnormalities of the genitourinary tract, in order to limit further confounding parameters. Patients with moderate–severe urogenital prolapse were excluded, as the degree of bladder-emptying and urinary obstruction and the possible coexistence of stress urinary incontinence could have altered the final data analysis.

All enrolled patients were included into a final database.

All women enrolled in this study received three to four CO_2_ laser treatment sessions, each lasting 5 to 6 min, with an average period of 30 days between sessions. A complete course of treatment included at least 3 laser sessions, and a new complete cycle could be initiated after a minimum of 12 months. Local therapy was not recommended either before or after the laser sessions.

For the laser sessions the SmartXide2 V2 LR, Deka m.e.l.a., Florence, Italy fractional CO_2_ laser system was used in conjunction with a Vaginal Laser Reshaping (V2 LR) scanning system, and appropriate handpieces for the vaginal area. The following settings were applied: power of 40 W, scan time of 1000 ms, spacing of 1000 mm, and Smart stack ½ for vaginal laser; and power of 25 W, scan time of 500 ms, spacing of 500 mm, and Smart stack ½ for vulvar laser. This treatment approach is based on the interaction between the vaginal mucosa and a specific CO_2_ pulsed laser. A fractionated laser beam emitted by the CO_2_ laser was directed onto small spots spaced apart by healthy tissue. Each pulse delivers continuous high-energy peak power to quickly ablate the epithelial component of the vaginal mucosa. This is followed by prolonged emission periods, allowing the CO_2_ to penetrate deeper in the mucosa. The pulses are delivered by a specialized handpiece emitting energy at 360° pattern. Following the initial energy release, the handpiece is rotated clockwise by approximately 2 cm while maintaining the same vaginal distance. During all treatment sessions, a speculum is positioned for a vaginal examination in the preliminary phase, after which the handpiece is carefully inserted deep into the vaginal canal. The laser emissions are divided into various laser spots while progressively extracting the handpiece from the vaginal fundus to cover as much vaginal mucosa as possible, especially at the level of the anterior wall.

Before starting the sessions, all women underwent a complete gynecological examination, including pelvic transvaginal ultrasound, urinalysis, and urinary cytology sampling. A visual analog scale (VAS) evaluation, ranging from 0 to 10, was administered to assess baseline urinary symptoms associated with cystitis such as dysuria, daily pollakiuria and urgency. Additionally, the enrolled patients were asked to report in a diary the number of cystitis episodes experienced in the previous 12 months pre-treatment. An additional feature investigated within the gynecological examination was the presence or absence of vaginal atrophy according to the Vulva Health Index (VHI).

After completing the full cycle of CO_2_ laser treatment, all women underwent a follow-up examination 12 months after the initial laser session and a post-treatment evaluation using the VAS (ranging from 0–10) to evaluate dysuria, daily pollakiuria and urgency. Additionally, the enrolled patients were asked to indicate the number of cystitis episodes experienced during the 12 months following treatment.

R software (version 4.3.1) was utilized for all statistical analyses. In particular, the stats package was used to conduct non-parametric Wilcoxon tests. The choice of the Wilcoxon test was dictated by the nature of the obtained data, which did not adhere to a normal distribution, as confirmed by a Shapiro–Wilk test. The adoption of non-parametric tests is appropriate for data that do not meet assumptions of normality, providing a robust alternative to parametric tests such as the t-test. All statistical tests were two-tailed and evaluated at a significance level α ≤ 0.05. The Shapiro–Wilk test was performed to assess normality of the data distributions for the primary outcome measures (dysuria, daily pollakiuria, and urgency scores). The results indicated significant deviations from normality (*p* ≤ 0.05), justifying the use of non-parametric methods. The Wilcoxon test was then applied to compare pre-treatment and post-treatment scores within the same group of patients. The test is particularly well-suited to our paired data because it accounts for the non-normal distribution and is effective in analyzing changes in median values over time. By using Wilcoxon’s non-parametric test, we ensured that the analysis remained valid and reliable despite the non-normal data distribution, thus providing a more accurate assessment of treatment effects.

## 3. Results

A total number of 240 women referring to Fondazione Policlinico Universitario Campus Bio Medico of Rome and Ospedale di Stato of St. Marino Republic with interstitial, post-coital and recurrent cystitis were collected. A total of 124 women were excluded for the following reasons: 26 patients due to previous gynecological surgery, 12 due to previous pelvic radiation therapy, 15 due to uncontrolled diabetes mellitus and hypertension, 18 due to urinary tract neoplasms, 24 due to ongoing pharmacological therapy for urge incontinence, 19 due to urinary tract abnormalities and genital prolapse greater than grade 2, and 10 due to ongoing urinary tract infections.

**Figure 1:**
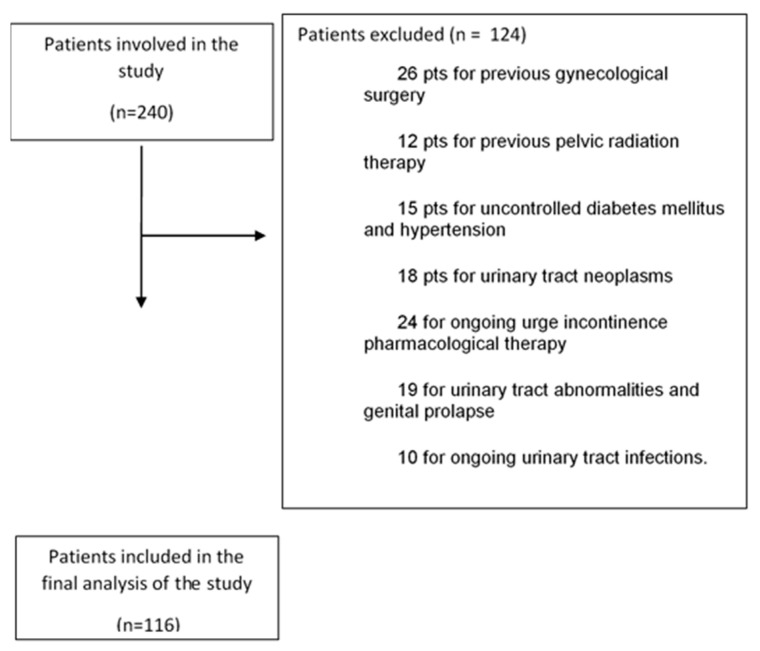
Study flow chart.

Based on the inclusion and exclusion criteria, the study enrolled a total of 116 pre- and post-menopausal women for final analysis (Figure 1). The average age of the participants was 54 years (27–75). The population included 70 (60.3%) menopausal women and 46 (39.7%) premenopausal women. None of the pre-menopausal women were undergoing therapy with combined estrogen–progestogen pills. Conversely, among the menopausal women, a total of 19 patients (27% of the court and 16% of the total population under examination) were receiving hormone replacement therapy (HRT). It was observed that 36 patients (31%) did not receive any other local therapy during the entire laser therapy cycle, while 20 patients (17%) underwent a concurrent weekly session of intravesical instillation of hyaluronic acid, and 60 patients (47%) used daily local applications of hyaluronic acid cream throughout the laser cycle (Table 1).

Within the entire study population, 5 patients (4.5%) were diagnosed with interstitial cystitis, 77 patients (66%) were diagnosed with recurrent cystitis, 29 patients (25%) were diagnosed with post-coital cystitis and 5 patients (4.5%) were diagnosed with both post-coital cystitis and recurrent cystitis. Table 2 shows the number and types of cystitis within each specific sub-population under examination (Table 2).

Among the entire population it was observed that in the 12 months prior to treatment, the average number of cystitis episode per year within the study cohort was five episodes (range: 3–11). Following completion of a full course of laser treatment (comprising 3 to 4 sessions), the average number of cystitis episodes observed in the entire population during the subsequent 12 months was 1.7 (range: 0 to 5). Table 3 shows the number of acute cystitis episodes observed within each specific sub-population over the course of one year in the research study (Table 3).

**Figure 2:**
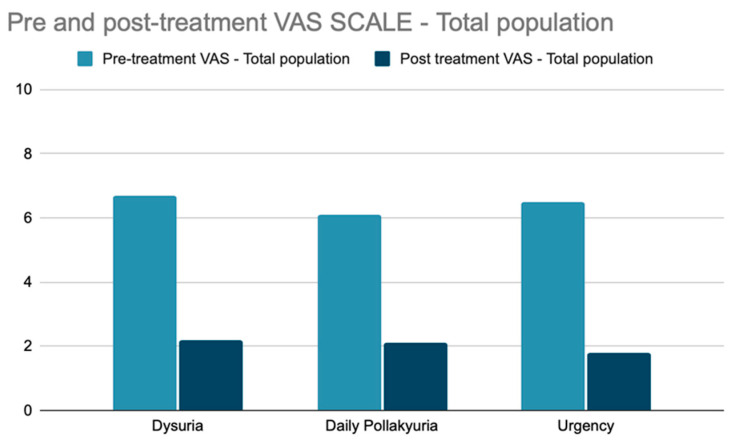
Pre- and post-treatment VAS scale assessment for dysuria, daily pollakiuria and urgency in the whole population.

The VAS scale administered for the initial assessment of pre-treatment dysuria, pollakiuria, and urgency showed the following results: the mean VAS for dysuria was 6.9/10, the mean VAS for pollakiuria was 6.3/10, and the mean VAS for urgency was 6.6/10 (Figure 2). The VAS scale administered for the assessment of the residual degree of post-treatment dysuria, pollakiuria, and urgency showed the following results: the mean VAS for dysuria was 1.9/10 (*p*-value: <2.2 × 10^−16^), the mean VAS for pollakiuria was 1.6/10 (*p*-value: <2.2 × 10^−16^), and the mean VAS for urgency was 1.6/10 (*p*-value: <2.2 × 10^−16^).

**Figure 3:**
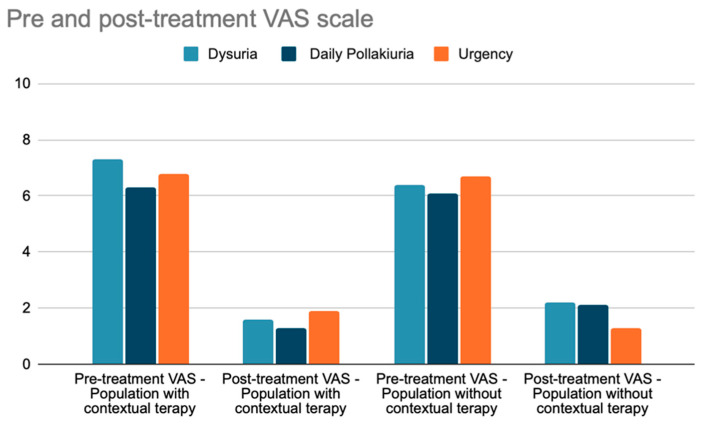
Pre- and post-treatment VAS scale assessment for dysuria, daily pollakiuria, and urgency in the subgroups with and without concomitant hyaluronic acid therapy.

A further investigation utilizing the VAS scale was carried out within the subgroup of individuals who had undergone intravesical hyaluronic acid therapy alongside vaginal laser treatment (Figure 3). The mean VAS for dysuria among patients who underwent contextual therapy before treatment was 7.3/10; after treatment, it was 1.6/10. For the group of patients who did not undergo contextual therapy, the mean VAS for dysuria before treatment was 6.4/10, and after treatment, it was 2.2/10 (*p*-value: 0.0006764). The mean VAS for urgency among patients who underwent contextual therapy before treatment was 6.8/10; after treatment, the mean VAS was 1.9/10. For those patients who did not undergo contextual therapy, the VAS scale for urgency before treatment was 6.7/10, and after treatment, it was 1.3/10 (*p*-value: 0.2897). The mean VAS for pollakiuria among patients who underwent contextual therapy before treatment was 6.3/10, and post-treatment, it was 1.3/10. In the group of patients who did not undergo contextual therapy, the mean VAS scale for pollakiuria before treatment was 6.1/10, and after treatment, it was 2.1/10 (*p*-value: 0.000308).

**Figure 4:**
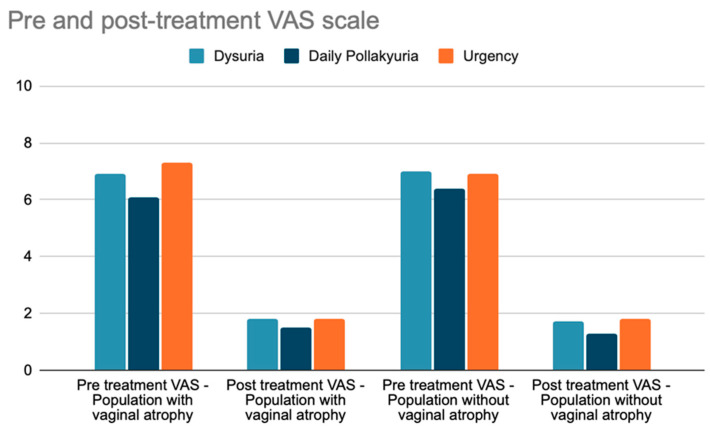
Pre- and post-treatment VAS scale assessment for dysuria, daily pollakiuria, and urgency in the subgroups with and without vaginal atrophy.

The VAS scale improved in both groups of patients, whether they had vaginal atrophy or not (Figure 4). The VAS scale for dysuria in patients with vaginal atrophy before treatment was 6.9/10, and after treatment it was 1.8/10. The VAS scale for urgency in patients with vaginal atrophy before treatment was 7.3/10, and after treatment, it was 1.8/10. The VAS scale for pollakiuria in patients with vaginal atrophy before treatment was 6.1/10, and after treatment, it was 1.5/10. The VAS scale for dysuria in patients without vaginal atrophy before treatment was 7.0/10, and after treatment, it was 1.7/10. The VAS scale for urgency in patients without vaginal atrophy before treatment was 6.9/10, and after treatment, it was 1.8/10. The VAS scale for pollakiuria in patients without vaginal atrophy before treatment was 6.4/10, and after treatment, it was 1.3/10. Both groups of patients with vaginal atrophy and without vaginal atrophy showed a positive outcome after the treatment, there were no side effects during the therapy; the *p*-value for dysuria was 0.6495, the *p*-value for urgency was 0.5325, and the *p*-value for pollakiuria was 0.2496. No side or adverse effects were reported from patients at the 12-month follow-up gynecological examination.

## 4. Discussion

This is the first multicentric prospective study analyzing the efficacy and effectiveness of the micro-ablative vaginal CO2 laser on interstitial, post-coital and recurrent cystitis in pre- and post-menopausal women for the management of dysuria, urgency, and daily pollakiuria. The strength of this study is its multicentric prospective nature, including multiple measures related to different aspects of cystitis.

Within the current scientific literature, numerous studies have investigated the effectiveness of laser treatment in managing urogenital symptoms. This therapy has been widely explored and applied across various subspecialties within the fields of functional urology and uro-gynecology. The laser is, in fact, a valuable approach for treating a wide range of functional diseases, such as interstitial cystitis, trigonitis, mesh complications, stress/urge incontinence and pelvic organ prolapse. The availability of numerous types of lasers and application techniques has greatly enhanced the efficacy and versatility of this treatment method [39,40]. A 2022 prospective study demonstrated the efficacy of fractional micro-ablative vaginal CO2 laser therapy in treating urinary incontinence. The study showed its effectiveness, particularly in women with post-menopausal vaginal atrophy wherein the VAS scale indicated significant improvement among patients with either SUI or MUI [41]. Moreover, another previous retrospective study published by Angioli et al., including 165 post-menopausal women with vulvo–vaginal atrophy and gynecological cancers, demonstrated the efficacy of micro-ablative CO_2_ laser treatment in ameliorating symptoms such as dryness, dyspareunia, burning, pain at introitus, and itching, thereby significantly enhancing the quality of life of the patients [42].

Although lasers potentially provide advantages over other therapy strategies, such as in the case of interstitial cystitis and GSM, their adoption has been discontinued in some of the most common uro–gynecological pathologies. Moreover, scientific studies involving laser therapy often have a limited number of patients and relatively short follow-up times. In certain cases, there is insufficient evidence to support the safety and effectiveness of laser as substitutes for traditional first-line therapies. However, their low invasiveness and ease of administration do open the possibility for further investigations regarding their application to a wider range of patients [43,44].

This prospective multicenter study represents the first investigation in the literature to explore the efficacy of micro-ablative CO_2_ vaginal lasers for managing urogenital symptoms across a large and diverse population. The study includes participants in both pre- and post- menopause, with or without vaginal atrophy, and undergoing or not undergoing concurrent local therapy. It was decided to include a sub-group of pre-menopausal patients to evaluate the effectiveness of the micro-ablative vaginal laser on a population that has poorly been analyzed previously in the scientific literature. Over the years, numerous studies have investigated the effectiveness of vaginal lasers on local disorders associated with hormonal deficiency in menopause, adopting them for the management of GMS, vulvo–vaginal atrophy and even for the postoperative complications of uro–gynecological surgery. At the same time, there is not much research related to a broader field of use for vaginal lasers such as on younger populations or inflammatory pathologies of the lower urinary tract. This study fits into this context.

The study primarily focused on individuals with recurrent and post-coital cystitis, with 77 patients and 29 patients affected, respectively, or a combination of both conditions (5 patients). Only five patients were diagnosed with histologically determined interstitial cystitis. It was decided to conduct the main statistical analysis on the entire study population, followed by a comparative sub-analysis dividing the participants into two distinct groups, as follows: patients with and without vaginal atrophy, and patients receiving and not receiving intra-vesical instillations of hyaluronic acid during the laser treatment cycle. This subdivision aimed to investigate how laser therapy affected more specific subsets of population with particular characteristics that might enhance the expected effects on local tissues. Specifically, it aimed to investigate whether the presence of vaginal atrophy or the concomitant administration of hyaluronic acid could modify the efficacy of micro-ablative CO_2_ laser therapy in managing urogenital symptoms.

In the overall study population, a complete treatment consisting of 3–4 monthly laser sessions resulted in a statistically significant reduction in the annual frequency of cystitis episodes reported by patients during the 12-month follow-up after the final treatment session (*p* value < 2.2). Applying the VAS scale before treatment initiation (T0) and at 12 months post-treatment (T1), statistically significant differences were observed across all three of the parameters assessed: dysuria, urgency and daytime pollakiuria (*p* value < 2.2 × 10^16^). A notable strength of this study lies in this longitudinal approach, enabling comprehensive follow-up assessments conducted one-year post-treatment. This extended timeframe enabled the thorough evaluation of laser therapy efficacy and patient-reported outcomes over time, providing a comprehensive understanding of the long-term effects of laser therapy; this study underscores the importance of considering patient perspectives beyond the immediate post-treatment period. The very low *p*-value, below 2.2 × 10^−16^, indicates the presence of strong statistical evidence that the observed changes are attributable to the treatment rather than chance. These findings highlighted the significant impact of treatment on symptoms evaluated using the VAS scale. The post-treatment reduction in dysuria, urgency, pollakiuria and the overall decrease in annual cystitis episodes suggests an improvement in patient condition and quality of life. These highlighted data are consistent with those reported in the UNICORN-2 and UNICORN-3 studies [45,46], despite their smaller sample sizes (12 and 15 patients, respectively), exclusively affected by interstitial cystitis/BPS and vulvodynia. Both of the aforementioned studies adopted non-ablative erbium and neodymium yttrium aluminum garnet (YAG) lasers.

A further sub-analysis was conducted by dividing the general population into two distinct groups: one subgroup concurrently undergoing weekly therapy with intra-vesical instillations of hyaluronic acid, and another subgroup not receiving any contextual therapy. The aim was to investigate whether there was a benefit from combining these two treatments.

When analyzing the results obtained, an interesting observation emerged regarding the effectiveness of contextual therapy in modifying symptoms between the two groups of patients. For dysuria and pollakiuria, the calculated *p*-value were notably low (0.0006764 and 0.000308, respectively), indicating a significant difference between the two groups of patients. Those who received concurrent therapy experienced a change in dysuria and pollakiuria to a significantly greater extent than those who did not receive such therapy. However, when considering urgency, the *p*-value (0.2897) suggests that there is no statistically significant difference between the two patient groups. These data suggest that concomitant therapy could be associated with better management of these two specific symptoms of cystitis and would therefore make it possible to select specific categories of patients benefiting most from laser treatments. Remarkably, this study is the first of its kind to examine the effectiveness of the vaginal laser as an adjuvant therapy alongside existing first-line treatments for various types of cystitis, especially recurrent ones, extending beyond interstitial/BPS. Recurrent cystitis significantly impacts the quality of life for affected patients and, due to its high prevalence in the general population, represents a substantial economic health burden in terms of pharmacological therapies, instrumental diagnostics, and specialist medical consultations [2,47,48].

In the second sub-analysis of this study, the population was further divided into two additional groups: pre- and post-menopausal women affected by vaginal atrophy and those not affected. Both groups exhibited a predominance of recurrent cystitis, but there was a difference in the distribution of other types of cystitis. Specifically, it was highlighted that patients without vaginal atrophy had a higher percentage of post-coital cystitis compared to those with vaginal atrophy. However, in both groups, a significant reduction in the average number of cystitis episodes per year after treatment was observed (*p* value < 2.2). Moreover, the group with vaginal atrophy shows a slightly higher average number of post-treatment cystitis episodes per year compared to the group without atrophy. This result is in contrast with the initial working hypothesis, according to which the micro-ablative vaginal laser, having demonstrated effectiveness in tissue regeneration, should have determined a more incisive reduction in the annual number of cystitis episodes in the population of atrophic patients. This sub-group, starting from a more deteriorated tissue substrate, should have benefited more from the laser’s regenerative effect, with a lower number of post-treatment relapses.

The analysis of the VAS scale shows a reduction in symptoms before and after treatment in both groups (*p* values: 2.2 × 10^−16^), with no significant differences in *p*-value values between patients without and with vulvovaginal atrophy. This suggests that both groups benefit similarly from the treatment, with no evidence suggesting greater efficacy of the laser in patients with vulvovaginal atrophy. This finding contrasts with the initial hypothesis of the study, which anticipated greater efficacy of the laser in atrophic patients. The initial hypothesis was justified by several studies in the literature, including a meta-analysis by Filippini et al. in 2022. This study highlighted the effectiveness of various lasers in alleviating urogenital menopausal symptoms in patients affected by vulvo–vaginal atrophy (VVA) or GSM [49]. Current studies in the literature suggest that vaginal laser therapy, with either the erbium-laser or the CO_2_ laser, is an effective therapeutic option to treat GSM symptoms, mainly for sexually related and urologic symptoms [50,51].

Summarizing and correlating the initial hypotheses of the investigation with the results obtained, it was shown that: improvements were identified on all three genitourinary symptoms examined (daytime pollakiuria, dysuria and urgency) among the entire population (both pre- and post-menopausal women). The very low *p*-value, below 2.2 × 10^−16^, indicates strong statistical evidence that the observed changes are not due to chance, but probably attributable to the treatment itself. Moreover, it was demonstrated that a statistically significant reduction in the annual number of cystitis relapses occurred. In relation to the sub-population who underwent contextual intravesical hyaluronic acid therapy, the initial hypothesis was that it could achieve a more evident improvement in all urogenital symptoms examined, compared to the sub-population without concurrent intravesical therapy. The descriptive statistic confirms that both groups (patients with and without concurrent hyaluronic acid therapy) showed improvements in symptoms after treatment. However, patients who received concurrent therapy appear to have experienced a greater reduction in symptoms of dysuria and pollakiuria compared with patients without such treatment. This suggests that contextual therapy could be associated with better management of these specific symptoms in cystitis. In relation to the vaginal atrophy subpopulation, the initial working hypothesis supported the idea that these patients could benefit more from the laser therapy in terms of the management of urogenital symptoms compared to the population without vaginal atrophy. In fact, the VAS scale showed a reduction in pre- and post-treatment symptoms in both groups, but there were no significant differences in *p*-value values between patients without and with vulvovaginal atrophy. This suggests that both groups benefit similarly from the treatment, without the presence or absence of atrophy modifying the response to laser treatment. In addition, in both groups, a significant reduction in the average number of cystitis episodes per year after treatment was observed. However, the vulvovaginal atrophy group appears to have had a slightly higher average number of post-treatment cystitis episodes per year than the group without atrophy.

## 5. Conclusions

In conclusion, despite the small size of the study group and the varied concurrent therapies received by patients, the highlighted findings suggest that fractional CO_2_ laser therapy represents a safe and efficacious, non-hormonal approach for pre- and post-menopausal women diagnosed with recurrent, post-coital, and interstitial cystitis. The present study obtained notable results regarding the efficacy of the micro-ablative CO_2_ vaginal laser in improving of symptoms, such as dysuria, daytime pollakiuria and urinary urgency, in patients affected by interstitial, post-coital and recurrent infective and non-infective cystitis. Additionally, the treatment showed a reduction in the number of acute episodes on an annual basis. All this translates into a clear improvement in the quality of life for patients suffering from this debilitating pathology. In particular, the demonstrated superior efficacy in symptom management among patients undergoing intravesical instillations of hyaluronic acid could lay the foundations for the introduction of micro-ablative CO_2_ vaginal laser therapy in the treatment of cystitis, as a support for the therapies currently adopted. The clinical implications associated with the results obtained are numerous. The CO_2_ micro-ablative vaginal laser demonstrates an excellent applicability in an outpatient setting, with easy management, a good cost-effective ratio, notable compliance and tolerability for patients, and no short- or long-term undesirable effects. The treatment is rapid, manageable, and painless and the monthly application allows for good patient adherence to the therapeutic program.

Further studies, perhaps involving a larger sample size and conducted over a longer duration, will be required to effectively translate the demonstrated efficacy and symptom management benefits into the incorporation of CO_2_ vaginal lasers as a valuable therapeutic option for treating these uro–gynecological pathologies. A larger sample may include women of different ages in order to correlate the effectiveness of the treatment with each age group’s specific risk factors and demographic characteristics and identify patients who would benefit most from the therapy. This research should also evaluate potential risks and both short-/long-term benefits associated with its use; therefore. a longer follow-up period could allow for a more complete investigation. Another aspect that needs to be addressed is the cost-effectiveness of lasers, especially from a longer-term viewpoint, considering that their adoption requires periodical re-application.

## Figures and Tables

**Table 1:** Population demographic characteristics.

	Numbers	Mean Age
Total nr of patients	116	54 (27–75)
Pre-menopausal patients	46 (39.7%)	37 (21–45)
Post-menopausal patients	70 (60.3%)	57 (44–75)
Patients with contextual intravesical hyaluronic acid therapy	81 (70%)	56 (27–76)
Patients without contextual intravesical hyaluronic acid therapy	35 (30%)	57 (36–72)
Patients with vulvo–vaginal atrophy	50 (34.5%)	50 (27–72)
Patients without vulvo–vaginal atrophy	66 (65.5%)	61 (36–76)

**Table 2:** Population demographic characteristics.

	Pts with Interstitial Cystitis	Pts with Recurrent Cystitis	Pts with Post-Coital Cystitis	Pts with Recurrent and Post-Coital Cystitis
Patients with contextual intravesical hyaluronic acid therapy	1 (1.2%)	49 (60.5%)	23 (28.4%)	1 (1.2%)
Patients without contextual intravesical hyaluronic acid therapy	2 (5.7%)	23 (65.7%)	6 (17.1%)	3 (8.6%)
Patients with vaginal atrophy	1 (1.5%)	54 (81.8%)	6 (9.1%)	2 (3.0%)
Patients without vaginal atrophy	2 (4.0%)	18 (36.0%)	23 (46.0%)	3 (6.0%)
Total nr of patients	5 (4.5%)	77 (66%)	29 (25%)	5 (4.5%)

**Table 3:** Pre- and post-treatment cystitis episodes per year.

	Pre-Treatment Average Number of Cystitis Episodes per Year	Pos-Treatment Average Number of Cystitis Episodes per Year
Patients with contextual intravesical hyaluronic acid therapy	4.9 (3–11)	1.7 (0–5)
Patients without contextual intravesical hyaluronic acid therapy	4.8 (3–9)	1.1 (0–3)
Patients with vaginal atrophy	5.4 (3–11)	2.4 (0–5)
Patients without vaginal atrophy	5.1 (3–8)	1.7 (0–4)
Total nr of patients	5.1 (3–9)	2.4 (0–7)

## Data Availability

Data are unavailable due to privacy.
